# Genome sequence and description of *Haloferax massiliense* sp. nov., a new halophilic archaeon isolated from the human gut

**DOI:** 10.1007/s00792-018-1011-1

**Published:** 2018-02-12

**Authors:** Saber Khelaifia, Aurelia Caputo, Claudia Andrieu, Frederique Cadoret, Nicholas Armstrong, Caroline Michelle, Jean-Christophe Lagier, Felix Djossou, Pierre-Edouard Fournier, Didier Raoult

**Affiliations:** 1 Unité de Recherche sur les Maladies Infectieuses et Tropicales Emergentes, CNRS, (UMR 7278) IRD (198), INSERM (U1095), AMU (UM63), Institut Hospitalo-Universitaire Méditerranée-Infection, Aix-Marseille Université, 19-21 Boulevard Jean Moulin, 13385 Marseille Cedex 5, France; 2 Infectious and Tropical Diseases Department, Centre Hospitalier Andrée-Rosemon, Cayenne, French Guiana; 3 Special Infectious Agents Unit, King Fahd Medical Research Center, King Abdulaziz University, Jeddah, Saudi Arabia

**Keywords:** Culturomics, Taxono-genomics, Halophilic archaea, *Haloferax massiliense*

## Abstract

By applying the culturomics concept and using culture conditions containing a high salt concentration, we herein isolated the first known halophilic archaeon colonizing the human gut. Here we described its phenotypic and biochemical characterization as well as its genome annotation. Strain Arc-Hr^T^ (= CSUR P0974 = CECT 9307) was mesophile and grew optimally at 37 °C and pH 7. Strain Arc-Hr^T^ was also extremely halophilic with an optimal growth observed at 15% NaCl. It showed gram-negative cocci, was strictly aerobic, non-motile and non-spore-forming, and exhibited catalase and oxidase activities. The 4,015,175 bp long genome exhibits a G + C% content of 65.36% and contains 3911 protein-coding and 64 predicted RNA genes. PCR-amplified 16S rRNA gene of strain Arc-Hr^T^ yielded a 99.2% sequence similarity with *Haloferax prahovense*, the phylogenetically closest validly published species in the *Haloferax* genus. The DDH was of 50.70 ± 5.2% with *H. prahovense*, 53.70 ± 2.69% with *H. volcanii*, 50.90 ± 2.64% with *H. alexandrinus*, 52.90 ± 2.67% with *H. gibbonsii* and 54.30 ± 2.70% with *H. lucentense*. The data herein represented confirm strain Arc-Hr^T^ as a unique species and consequently we propose its classification as representative of a novel species belonging to the genus *Haloferax*, as *Haloferax massiliense* sp. nov.

## Introduction

The human intestinal microbiota is a complex ecosystem consisting of a wide diversity including bacteria (Lagier et al. 2012), archaea (Khelaifia et al. 2013), and unicellular eukaryotes (Nam et al. 2008). The culturomics concept, recently introduced in our laboratory to study the prokaryotes diversity in the human gut (Lagier et al. 2012), allowed the isolation of a huge halophilic bacteria diversity including several new species (Lagier et al. 2016). Among the diverse culture conditions and several culture media used by culturomics to isolate new prokaryotes, some conditions targeting specifically extremophile organisms were also used (Lagier et al. 2016). Indeed, culture media containing high salt concentration are essentially used to select halophilic bacteria and archaea.

Currently, the determination of the affiliation of a new prokaryote is based on the 16S rDNA sequence, G + C content % and DNA–DNA hybridization (DDH). This approach is limited because of the very low cutoff between species and genera (Welker and Moore 2011). In some cases, 16S rRNA gene sequence comparison has been proved to poorly discriminate some species belonging to a same genus and remain ineffective (Stackebrandt and Ebers 2006). Recently, we proposed a polyphasic approach based on phenotypic and biochemical characterization, MALDI-TOF MS spectrum and total genome sequencing and annotation to better define and classify new taxa (Ramasamy et al. 2014).

Using culturomics techniques to isolate halophilic prokaryotes colonizing the human gut (Lagier et al. 2016), strain Arc-Hr^T^ was isolated from a stool specimen of a 22-year-old Amazonian obese female patient (Khelaifia and Raoult 2016). This strain presented different characteristics enabling its classification as a new species of the *Haloferax* genus. The *Haloferax* genus was first described by Torreblanca et al. (1986) and actually includes 12 species with validly published names. Members of the *Haloferax* genus are essentially extremely halophilic archaea that inhabit hypersaline environments such as the Dead Sea and the Great Salt Lake. They are classified in the family *Haloferacaceae* within the *Euryarchaeota* phylum and the various species constitute 57 recognized genera (Arahal et al. 2017).

In this study, we present a classification and a set of characteristics for *Haloferax massiliense* sp. nov., strain Arc-Hr^T^ (= CSUR P0974 = CECT 9307) with its complete genome sequencing and annotation.

## Materials and methods

### Ethics and samples collection

The stool specimens were collected from a 22-year-old Amazonian obese female patient after defecation in sterile plastic containers, sampled and stored at – 80 °C until use. Informed and signed consent was obtained from the patient. The study and the assent procedure were approved by the Ethics Committees of the IHU Méditerranée Infection (Faculty of Medicine, Marseille, France), under agreement number 09-022. Salt concentration of the stool specimen was measured by digital refractometer (Fisher scientific, Illkirch, France) and the pH was measured using a pH-meter.

## Isolation of the strain

Strain Arc-Hr^T^ was isolated in December 2013 by aerobic culture of the stool specimen in a home-made culture medium consisting of a Columbia broth (Sigma-Aldrich, Saint-Quentin Fallavier, France) modified by the addition of (per liter): MgCl_2_·6H_2_O, 15 g; MgSO_4_·7H_2_O, 20 g; KCl, 4 g; CaCl_2_·2H_2_O, 2 g; NaBr, 0.5 g; NaHCO_3_, 0.5 g, glucose, 2 g and 150 g of NaCl. pH was adjusted to 7.5 with 10 M NaOH before autoclaving. Approximately, 1 g of stool specimen was inoculated into 100 mL of this liquid medium in a flask incubated aerobically at 37 °C with stirring at 150 rpm. Subcultures were realized after 10, 15, 20 and 30 days of incubation. Then, serial dilutions of 10^−1^–10^−10^ were performed in the home-made liquid culture medium and then plated onto agar plates consisting of the previously detailed liquid medium with 1.5% agar.

### Strain identification by MALDI-TOF MS and 16S rRNA gene sequencing

MALDI-TOF MS protein analysis was carried out as previously described (Seng et al. 2013). The resulting 12 spectra of strain Arc-Hr^T^ were imported into the MALDI BioTyper software (version 2.0, Bruker) and analyzed by standard pattern matching (with default parameter settings) against the main spectra of halophilic and methanogenic archaea including the spectra from *Haloferax alexandrinus, Methanobrevibacter smithii, Methanobrevibacter oralis, Methanobrevibacter arboriphilus,* and *Methanomassilicoccus massiliensis*. The 16S rRNA gene amplification by PCR and sequencing were performed as previously described (Lepp et al. 2004). The phylogenetic tree was reconstructed according to the method described by Elsawi et al. (2017).

### Growth conditions

The optimum growth temperature of strain Arc-Hr^T^ was tested on the solid medium by inoculating 10^5^ CFU/mL of an exponentially growing culture incubated aerobically at 28, 37, 45***, and 55 °C. Growth atmosphere was tested under aerobic atmosphere, in the presence of 5% CO_2_, and also in microaerophilic and anaerobic atmospheres created using GENbag microaer and GENbag anaer (BioMérieux, Marcy l’Etoile, France) respectively. The optimum NaCl concentration required for growth was tested on solid media at 0, 1, 5, 7.5, 10, 15, 20, 25** and 30% of NaCl. The optimum pH was determined by growth testing at pH 5, 6, 7, 7.5, 8 and 9.

### Biochemical, sporulation and motility assays

To characterize the biochemical properties of strain Arc-Hr^T^, we used the commercially available Api ZYM, Api 20 NE, Api 50 CH strips (bioMérieux), supplemented by 15% NaCl (w/v) and 30 g/L of MgSO_4_. The sporulation test was done by thermic-shock at 80 °C for 20 min and subculturing on the solid medium. The motility of strain Arc-Hr^T^ was assessed by observing a fresh culture under DM1000 photonic microscope (Leica Microsystems, Nanterre, France) with a 100X oil-immersion objective lens. The colonies’ surface was observed on the agar culture medium after 3 days of incubation under aerobic conditions at 37 °C.

### Antibiotic susceptibility testing

Susceptibility of strain Arc-Hr^T^ to antibiotics was tested using antibiotic disks (B. Braun Medical SAS, Boulogne, France) containing the following antibiotics: fosfomycin 50 µg, doxycycline 30UI, rifampicin 30 µg, vancomycin 30 µg, amoxicillin 20 µg, erythromycin 15UI, ampicillin 25 µg, cefoxitin 30 µg, colistin 50 µg, tobramycin 10 µg, gentamicin 500 µg, penicillin G 10UI, trimethoprim 1.25 µg/sulfamethoxazole 23.75 µg, oxacillin 5 µg, imipenem 10 µg, metronidazole 4 µg and anisomycin 10 µg.

### Microscopy and gram test

Cells were fixed with 2.5% glutaraldehyde in 0.1 M cacodylate buffer for at least 1 h at 4 °C. A drop of cell suspension was deposited for approximately 5 min on glow-discharged formvar carbon film on 400 mesh nickel grids (FCF400-Ni, EMS). The grids were dried on blotting paper and cells were negatively stained for 10 s with 1% ammonium molybdate solution in filtered water at RT. Electron micrographs were acquired with a Morgagni 268D (Philips) transmission electron microscope operated at 80 keV. The gram stain was performed using the color GRAM 2 kit (Biomerieux) and observed using a DM1000 photonic microscope (Leica Microsystems).

### Analysis of fatty acid methyl ester and membrane polar lipids

Polar lipids were extracted and identified by one-dimensional TLC as described by Cui and Zhang (2014). Cellular fatty acid methyl ester (FAME) analysis was performed by GC/MS. Three samples were prepared with approximately 80 mg of bacterial biomass per tube harvested from several culture plates. Fatty acid methyl esters were prepared as described by Sasser (2006). GC/MS analyses were carried out as described before (Dione et al. 2016). Briefly, fatty acid methyl esters were separated using an Elite 5-MS column and monitored by mass spectrometry (Clarus 500—SQ 8 S, Perkin Elmer, Courtaboeuf, France). Spectral database search was performed using MS Search 2.0 operated with the Standard Reference Database 1A (NIST, Gaithersburg, USA) and the FAMEs mass spectral database (Wiley, Chichester, UK).

### DNA extraction and genome sequencing

After scraping 5 Petri dishes in 1 mL TE buffer, the genomic DNA (gDNA) of strain Arc-Hr^T^ was extracted from 200 µL of the bacterial suspension after a classical lysis treatment with a final concentration of lysozyme at 40 mg/mL for 2 h at 37 °C followed by an incubation time of 1 h at 37 °C in SDS 1% final and 30µL RNAse. Proteinase K treatment was realized with at 37 °C. After three phenol extractions and alcohol precipitation, the sample was eluted in the minimal volume of 50 µL in EB buffer. DNA was quantified by a Qubit assay with the high sensitivity kit (Life technologies, Carlsbad, CA, USA) to 14 ng/µL.

GDNA was sequenced on the MiSeq Technology (Illumina Inc, San Diego, CA, USA) with the mate pair strategy as previously described (Dione et al. 2016). Total information of 10.6 Gb was obtained from a 1326 K/mm^2^ cluster density with a cluster passing quality control filters of 99.1% (20,978,044 pass filter clusters). Within this run, the index representation for strain Arc-Hr^T^ was determined to be of 6.22%. The 1,303,974 paired reads were filtered according to the read qualities, trimmed and then assembled.

### Genome assembly

Illumina reads were trimmed using Trimmomatic (Lohse et al. 2012), then assembled thought Spades software (Nurk et al. 2013; Bankevich et al. 2012). Contigs obtained were combined together by SSpace (Boetzer et al. 2011) and Opera software (Gao et al. 2011) helped by GapFiller (Boetzer and Pirovano 2012) to reduce the set. Some manual refinements using CLC Genomics v7 software (CLC bio, Aarhus, Denmark) and homemade tools in Python improved the genome. Finally, the draft genome of strain Arc-Hr^T^ consisted of 8 contigs.

### Genome annotation and comparison

Non-coding genes and miscellaneous features were predicted using RNAmmer (Lagesen et al. 2007), ARAGORN (Laslett and Canback 2004), Rfam (Griffiths-Jones et al. 2003), PFAM (Punta et al. 2012), and Infernal (Nawrocki et al. 2009). Coding DNA sequences (CDSs) were predicted using Prodigal (Hyatt et al. 2010) and functional annotation was achieved using BLAST + (Camacho et al. 2009) and HMMER3 (Eddy 2011) against the UniProtKB database (The UniProt Consortium 2011). A brief genomic comparison was also made between strain Arc-Hr^T^ (CSTE00000000), *Haloferax alexandrinus* strain Arc-Hr (CCDK00000000), *Haloferax gibbonsii* strain ARA6 (CP011947), *Haloferax lucentense* strain DSM 14919 (AOLH00000000), *Haloferax volcanii* strain DS2 (CP001956) and *Haloferax prahovense* strain DSM 18310 (AOLG00000000). To estimate the mean level of nucleotide sequence similarity at the genome level between strain Arc-Hr^T^ and the four closest species with an available genome, we used the Average Genomic Identity of Orthologous gene Sequences (AGIOS), in a laboratory’s pipeline. Briefly, this pipeline combines the Proteinortho (Lechner et al. 2011) software (with the following parameters: *e* value 1*e*^−5^, 30% identity, 50% coverage and algebraic connectivity of 50%) for the detection of orthologous proteins between genomes compared pairwise, retrieves the corresponding genes and determines the mean percentage of nucleotide sequence identity between orthologous ORFs using the Needleman–Wunsch global alignment algorithm (Ramasamy et al. 2014). Strain Arc-Hr^T^ genome was locally aligned 2-by-2 using BLAT algorithm (Kent 2002; Auch et al. 2010) against each selected genomes previously cited and DNA–DNA hybridization (DDH) values were estimated by using the genome-to genome sequence comparison (Auch et al. 2010).

## Results

### Strain identification and phylogenetic analysis

**Fig. 1:**
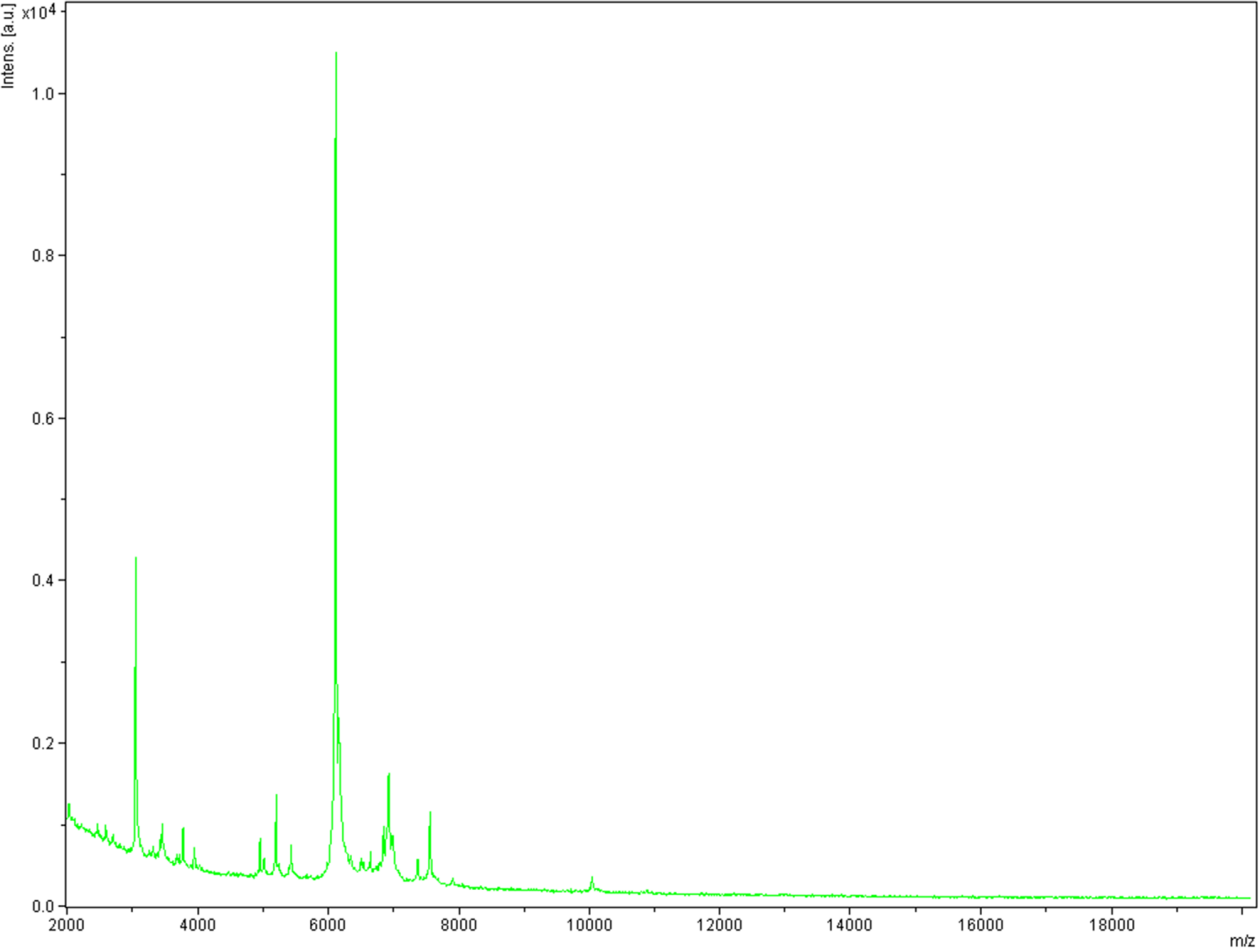
Reference mass spectrum from *Haloferax massiliense* strain Arc-Hr^T^. Spectra from 12 individual colonies were compared and a reference spectrum was generated

**Fig. 2:**
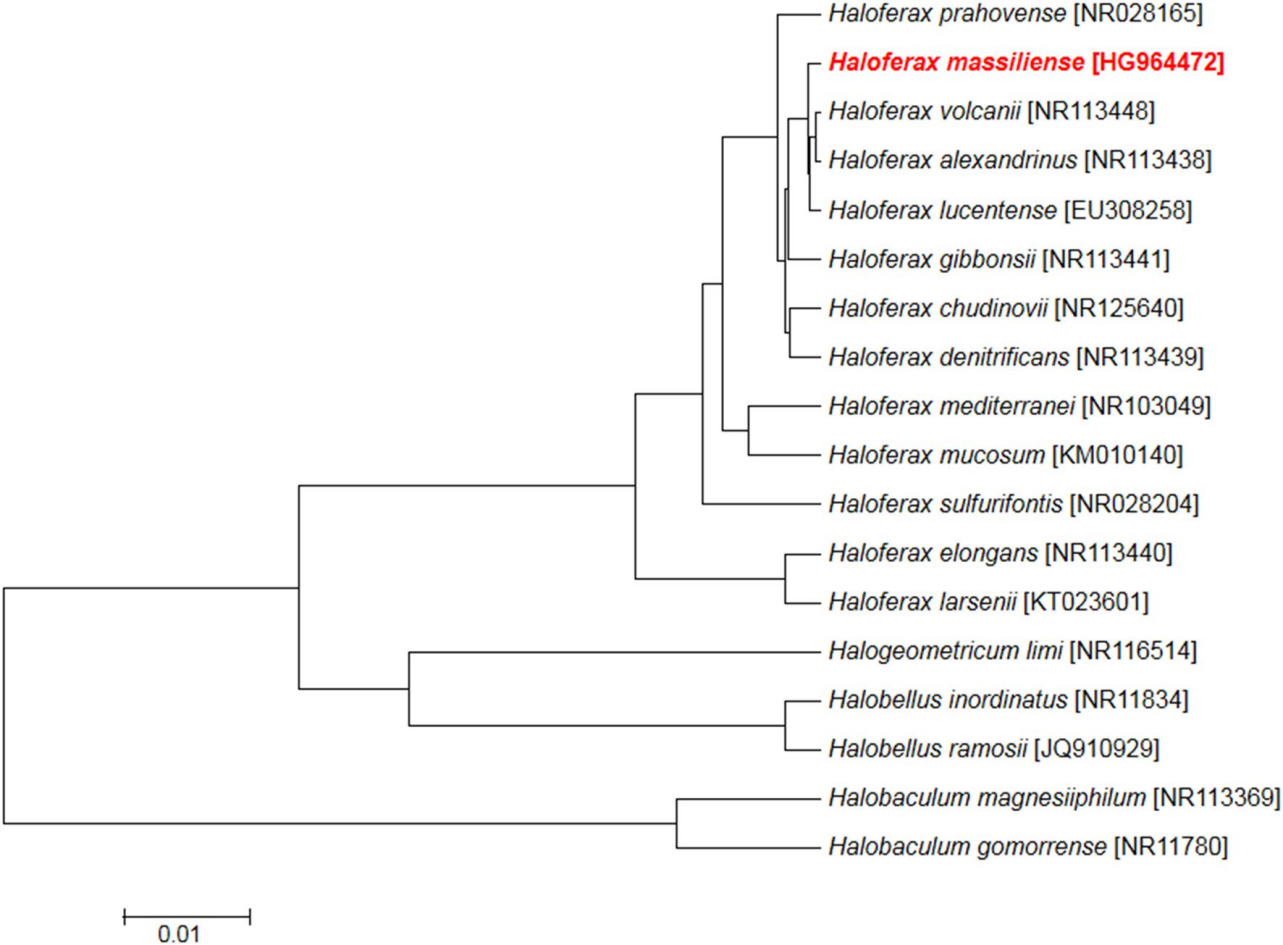
Phylogenetic tree highlighting the position of *Haloferax massiliense* strain Arc-Hr^T^ relative to other type strains within *Haloferax, Halogeometricum, Halobellus* and *Halobaculum* genus. The respective GenBank accession numbers for 16S rRNA genes are indicated in parenthesis. Sequences were aligned using CLUSTALW, and phylogenetic inferences were obtained using the maximum-likelihood method within the MEGA software. The scale bar represents 0.005% nucleotide sequence divergence

**Fig. 3:**
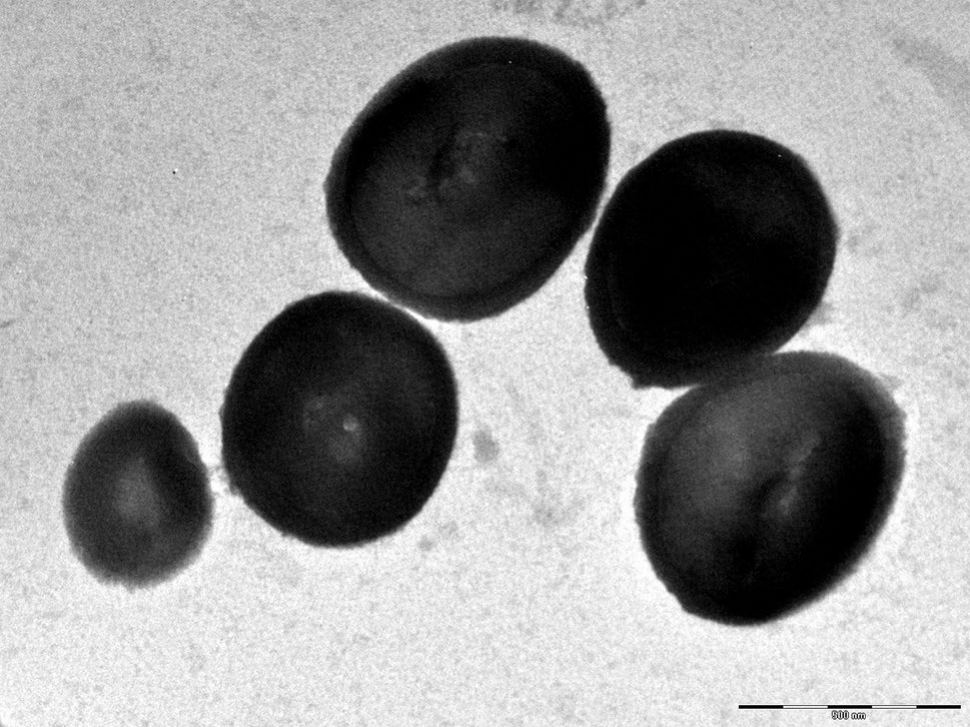
Transmission electron microscopy of *Haloferax massiliense* strain Arc-Hr^T^, using a Morgani 268D (Philips) at an operating voltage of 80 keV. The scale bar represents 500 nm

Using MALDI-TOF MS identification, no significant score allowing a correct identification was obtained for strain Arc-Hr^T^ against our database (the Bruker database is constantly incremented with URMITE data), suggesting that our isolate did not belong to any known species; and consequently, spectra from strain Arc-Hr^T^ was added to our database (http://www.mediterranee-infection.com/article.php?laref=256&titre=urms) (Fig. 1). PCR-amplified 16S rRNA gene of strain Arc-Hr^T^ (HG964472) exhibited a 99.2% sequence similarity with *Haloferax prahovense* JCM 13924 (NR113446), the phylogenetically closest validly published species with standing in nomenclature (Fig. 2). As 16S rRNA gene sequence comparison has been proven to poorly discriminate *Haloferax* species, we sequenced the complete genome of strain Arc-Hr^T^ and a digital DNA–DNA hybridization (dDDH) was made with four of the closest *Haloferax* species (see the part on genome comparison). These data confirmed strain Arc-Hr^T^ as a unique species. Finally, the gel view showed the protein spectral differences with other members of the genus *Haloferax* (Fig. 3).

### Phenotypic and biochemical characteristics

**Fig. 4:**
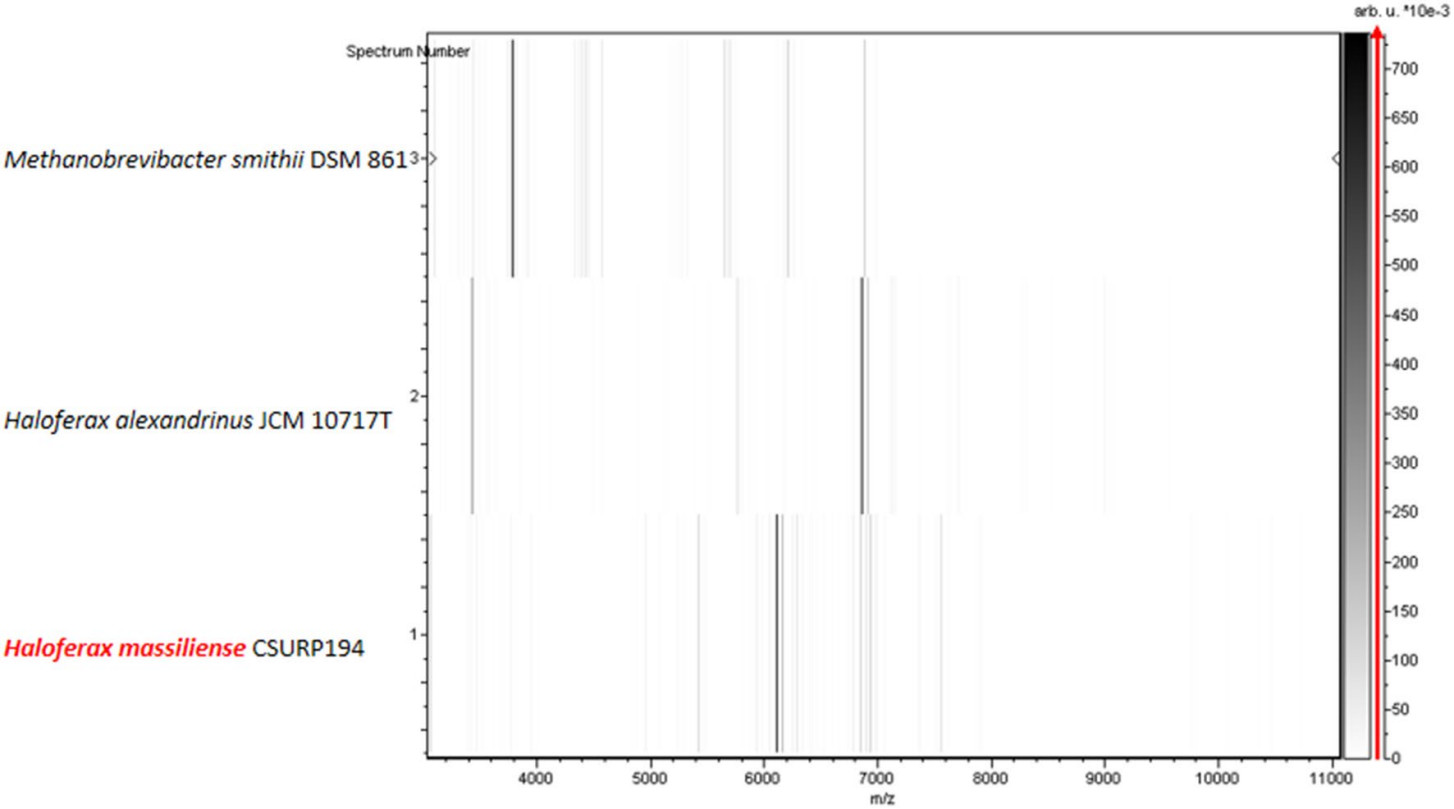
Gel view comparing *Haloferax massiliense* strain Arc-Hr^T^ to other species within the genus *Haloferax*. The gel view displays the raw spectra of loaded spectrum files arranged in a pseudo-gel like look. The *x*-axis records the *m*/*z* value. The left *y*-axis displays the running spectrum number originating from subsequent spectra loading. The peak intensity is expressed by a Gray scale scheme code. The color bar and the right *y*-axis indicate the relation between the color of a peak and the peak intensity, in arbitrary units. Displayed species are indicated on the left

Salt concentration of the stool specimen measured by digital refractometer was about 2.5% and the pH was 7.2. Strain Arc-Hr^T^ colonies were circular, red, shiny and smooth with a diameter of 0.5–1 mm. Cells were gram-negative, non-motile and non-spore-forming. Cells were very pleomorphic (irregular cocci, short and long rods, triangles and ovals) and had a diameter between 1 to 4 µm (Fig. 4). Strain Arc-Hr^T^ was mesophilic and grew at temperatures ranging from 25 to 45 °C, with an optimum at 37 °C. NaCl was required for growth and the strain grew at a salinity ranging from 10 to 25% of NaCl with an optimum at 15%; cells underwent lysis below 100 g/L NaCl. The optimum pH for growth was 7 (range between pH 6.5 and 8). The strain was strictly aerobic and grew in the presence of 5% CO_2_; no growth was observed in microaerophilic or anaerobic condition by using alternative electron acceptors such as nitrate or DMSO, or by fermenting l-arginine. Principal features are presented in Table 1.

**Table 1:** Classification and general features of *Haloferax massiliense* strain Arc-Hr^T^ according to the MIGS recommendations (Field et al. 2008)

MIGS ID	Property	Term	Evidence code^a^
	Current classification	Domain: *Archaea*	TAS (Woese et al. 1990)
		Phylum: *Euryarchaeota*	TAS (Cavalier-Smith 2002; Garrity and Holt 2001)
		Class: *Halobacteria*	TAS (Grant et al. 2001a, b; Gupta et al. 2015)
		Order: *Haloferacales*	TAS (Grant and Larsen 1989; Gupta et al. 2015)
		Family: *Haloferacaceae*	TAS (Grant and Larsen 1989; Gupta et al. 2015)
		Genus: *Haloferax*	TSA (Torreblanca et al. 1986)
		Species: *Haloferax massiliense*	IDA
		Type strain: Arc-Hr^T^	IDA
	Gram stain	negative	IDA
	Cell shape	Cocci	IDA
	Motility	Non motile	IDA
	Sporulation	Non spore-forming	IDA
	Temperature range	Mesophile	IDA
	Optimum temperature	37 °C	IDA
	pH	pH 6.5–8	
	Optimum pH	7	
MIGS-6.3	Salinity	10–25%	IDA
	Optimum salinity	15% NaCl	IDA
MIGS-22	Oxygen requirement	Strictly aerobic	IDA
	Carbon source	Unknown	IDA
	Energy source	Unknown	IDA
MIGS-6	Habitat	Human gut	IDA
MIGS-15	Biotic relationship	Free living	IDA
	Pathogenicity	Unknown	NAS
	Biosafety level	2	IDA
MIGS-14	Isolation	Human feces	IDA
MIGS-4	Geographic location	France	IDA
MIGS-5	Sample collection time	December 2013	IDA
MIGS-4.3	Depth	surface	IDA
MIGS-4.4	Altitude	0 m above sea level	IDA

^a^Evidence codes—IDA, Inferred from Direct Assay; TAS, Traceable Author Statement (i.e., a direct report exists in the literature); NAS, non-traceable Author Statement (i.e., not directly observed for the living, isolated sample, but based on a generally accepted property for the species, or anecdotal evidence). These evidence codes are from http://www.geneontology.org/GO.evidence.shtml of the Gene Ontology project (Ashburner et al. 2000). If the evidence is IDA, then the property was directly observed for a live isolate by one of the authors or an expert mentioned in the acknowledgements

Strain Arc-Hr^T^ exhibited positive catalase and oxidase activities. Using an API ZYM strip, positive reactions were observed for alkaline phosphatase, acid phosphatase, esterase (C4), esterase lipase (C8), leucine arylamidase, naphthol-AS-BI-phosphohydrolase, β-glucuronidase, and negative reactions were observed for lipase (C14), valine arylamidase, trypsin, α-chymotrypsin, β-galactosidase, *N*-acetyl- β-glucosaminidase, α-galactosidase, α-glucosidase, β-glucosidase, α-fucosidase, α-mannosidase. An API 50CH strip showed positive reaction for glycerol, d-fructose, l-rhamnose, potassium 2-ketogluconate and potassium 5-ketogluconate, and negative reactions for arbutin, salicin, d-maltose, d-sucrose, d-raffinose,, erythritol, d-ribose, d-xylose, l-xylose, d-adonitol, methyl-βd-xylopyranoside, d-glucose, d-galactose, d-lactose, l-sorbose, dulcitol, inositol, d-mannitol, d-sorbitol, methyl-αd-mannopyranoside, methyl-αd-glucopyranoside, d-cellobiose, d-melibiose, d-trehalose, d-melezitose, starch, glycogen, xylitol, gentiobiose, d-turanose, d-lyxose, d-tagatose, d-fucose, d-fucose, d-arabitol, d-arabitol***, and potassium gluconate. The phenotypic characteristics of strain Arc-Hr^T^ were compared with the most closely related species (Table 2).

**Table 2:** Differential characteristics of *Haloferax massiliense* strain Arc-Hr^T^, *Haloferax prahovense* (Enache et al. 2007), *Haloferax volcanii* (Torreblanca et al. 1986), *Haloferax denitrificans* (Tindall et al. 1989); 4, *Haloferax mediterranei* (Torreblanca et al. 1986), *Haloferax gibbonsii* (Juez et al. 1986)*, Haloferax alexandrinus* (Asker and Ohta 2002) and *Haloferax lucentense* (Gutierrez et al. 2002). na: No available data

Properties	*H. massiliense*	*H. prahovense*	*H. volcanii*	*H. denitrificans*	*H. gibbonsii*	*H. mediterranei*	*H. alexandrinus*	*H. lucentense*
Oxygen requirement	+	+	+	+	+	+	+	+
Gram stain	–	–	–	–	–	–	–	–
Salt requirement	+	+	+	+	+	+	+	+
Motility	–	–	–	–	–	+	–	+
Endospore formation	–	–	–	–	–	–	–	–
Indole	–	–	+	+	–	+	+	+
Tween 80 hydrolysis	+	+	+	–	–	+	+	+
Production of								
Alkaline phosphatase	+	na	na	na	na	na	+	na
Catalase	+	+	+	+	+	+	+	+
Oxidase	+	+	+	+	+	+	+	+
Nitrate reductase	+	–	+	+	–	+	+	–
Urease	–	na	na	–	na	na	–	na
β-galactosidase	–	na	+	+	+	na	+	–
*N*-acetyl-glucosamine	–	Na	na	na	na	na	+	na
Acid from								
l-Arabinose	–	–	+	na	+	+	+	+
Ribose	–	+	–	–	na	na	+	na
Mannose	–	–	–	–	+	+	–	na
Mannitol	–	–	na	na	na	+	na	–
Sucrose	–	–	+	+	+	+	+	–
d-Glucose	–	–	+	+	+	+	+	+
d-Fructose	+	+	+	+	+	+	+	+
d-Maltose	–	+	+	+	+	+	+	+
d-Lactose	–	+	–	–	–	+	–	–
Gelatin hydrolysis	–	–	–	+	+	+	+	–
Starch hydrolysis	–	+	–	–	–	+	–	–
Casein hydrolysis	+	–	–	–	+	+	–	–
Habitat	Human Gut	Salt lake	Bottom sedimen	solar saltern	Solar salterns	Solar salt pond	solar saltern	Water of a saltern

Antimicrobial susceptibility testing demonstrated that strain Arc-Hr^T^ was susceptible to rifampicin, trimethoprim/sulfamethoxazole and anisomycin, and resistant to fosfomycin, doxycycline, vancomycin, amoxicillin, erythromycin, ampicillin, cefoxitin, colistin, tobramycin, gentamicin, penicillin G, oxacillin, imipenem and metronidazole.

The fatty acid was 3-methyl-butanoic acid (5:0 iso), a branched short chain fatty acid. Phenylacetic acid, known as an antifungal agent (Ryan et al. 2009), was also detected. Membrane polar lipids were diglycosyl diether analogs of phosphatidylglycerol, phosphatidylglycerol, diglycosyl diether,**** and sulfated diglycosyl diether (S-DGD-1), the glycolipid marker of *Haloferax* spp. (Cui and Zhang. 2014). This fatty acid’s profile is completely different from that of bacteria described by (Dione et al. 2016).

### Genome sequencing information and annotation

**Fig. 5:**
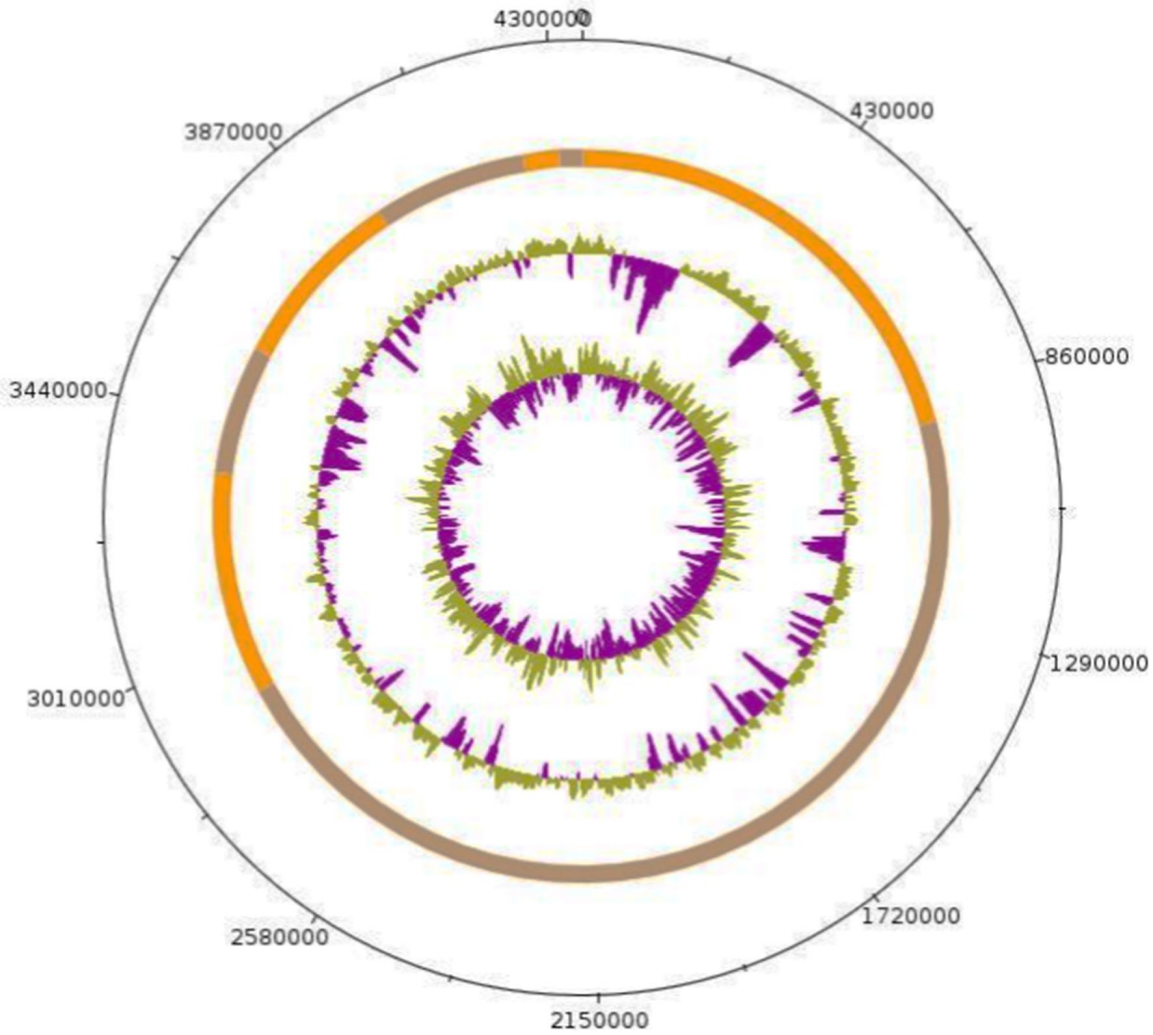
Circular representation of the *Haloferax massiliense* Arc-Hr^T^ genome. Circles from the center to the outside: GC screw (green/purple), GC content (green/purple) and contigs (orange/brown)

Strain Arc-Hr^T^’s genome was sequenced as part of a culturomic study aiming at isolating all prokaryotes species colonizing the human gut (Lagier et al. 2016) and because of its phylogenetic affiliation to the *Haloferax* genus. Strain Arc-Hr^T^ represents the 13th genome sequenced in the *Haloferax* genus. The draft genome of strain Arc-Hr^T^ contains 4,015,175 bp with a G + C content of 65.36% and consists of 8 contigs without gaps (Fig. 5). The genome was shown to encode at least 64 predicted RNA including 3 rRNA, 57 tRNA, 4 miscellaneous RNA and 3911 protein-coding genes. Among these genes, 490 (13%) were found to be putative proteins and 291 (8%) were assigned as hypothetical proteins. Moreover, 2335 genes matched at least one sequence in Clusters of Orthologous Groups (COGs) database (Tatusov et al. 1997, 2000) with BLASTP default parameters. Table 3 shows the detailed project information and its association with MIGS version 2.0 compliance. The properties and the statistics of the genome are summarized in Table 4. The distribution of genes into COGs functional categories is presented in Table 5.

**Table 3:** Project information

MIGS ID	Property	Term
MIGS-31	Finishing quality	High-quality draft
MIGS-28	Libraries used	1 mate-paired
MIGS-29	Sequencing platforms	MiSeq Illumina
MIGS-31.2	Sequencing coverage	620
MIGS-30	Assemblers	Spades
MIGS-32	Gene calling method	Prodigal
	Genbank ID	CSTE01000001–CSTE01000008
	Genbank date of release	Apr, 2014
MIGS-13	Source material identifier	Arc-Hr^T^
	Project relevance	Mar, 2014

**Table 4:** Nucleotide content and gene count levels of the genome

Attribute	Value	% of total^a^
Genome size (bp)	4,015,175	100
DNA coding region (bp)	3,414,159	78.50
DNA G + C content (bp)	2,624,318	65.36
Total protein-coding genes	3911	100
rRNA	3	0.08
tRNA	57	1.46
tmRNA	0	0
miscRNA	4	0.11
Genes with function prediction	2825	72.23
Genes assigned to COGs	3116	79.68

^a^The total is based on either the size of the genome in base pairs or the total number of protein coding genes in the annotated genome

**Table 5:** Number of genes associated with the 25 general COG functional categories

Code	Description	Value	% of total
J	Translation, ribosomal structure and biogenesis	165	4.22
A	RNA processing and modification	1	0.03
K	Transcription	172	4.40
L	Replication, recombination and repair	133	3.41
B	Chromatin structure and dynamics	6	0.16
D	Cell cycle control, cell division, chromosome partitioning	27	0.69
Y	Nuclear structure	0	0.0
V	Defense mechanisms	37	0.95
T	Signal transduction mechanisms	157	4.02
M	Cell wall/membrane biogenesis	119	3.05
N	Cell motility	38	0.98
Z	Cytoskeleton	0	0.0
W	Extracellular structures	0	0.0
U	Intracellular trafficking and secretion, and vesicular transport	35	0.90
O	Posttranslational modification, protein turnover, chaperones	113	2.89
C	Energy production and conversion	208	5.32
G	Carbohydrate transport and metabolism	219	5.6
E	Amino acid transport and metabolism	341	8.72
F	Nucleotide transport and metabolism	75	1.92
H	Coenzyme transport and metabolism	154	3.94
I	Lipid transport and metabolism	79	2.02
P	Inorganic ion transport and metabolism	202	5.17
Q	Secondary metabolites biosynthesis, transport and catabolism	54	1.39
R	General function prediction only	490	12.53
S	Function unknown	291	7.45

### Genome comparison

The draft genome of strain Arc-Hr^T^ is larger than that of *H. prahovense*, *H. alexandrinus, H. gibbonsii, H. lucentense* and *H. volcanii* (4.35, 4, 3.9, 3.62, 2.95 and 2.85 Mb respectively). The G + C content of strain Arc-Hr^T^ is smaller than that of *H. alexandrinus, H. lucentense, H. volcanii* and *H. gibbonsii* (65.36, 66, 66.4, 66.6 and 67.1%, respectively) but smaller than that of *H. prahovense* (65.7%). The gene content of strain Arc-Hr^T^ is larger than that of *H. alexandrinus, H. prahovense, H. lucentense, H. gibbonsii* and *H. volcanii* (3.911, 3.770, 3.766, 3.593, 2.997 and 2.917).

**Fig. 6:**
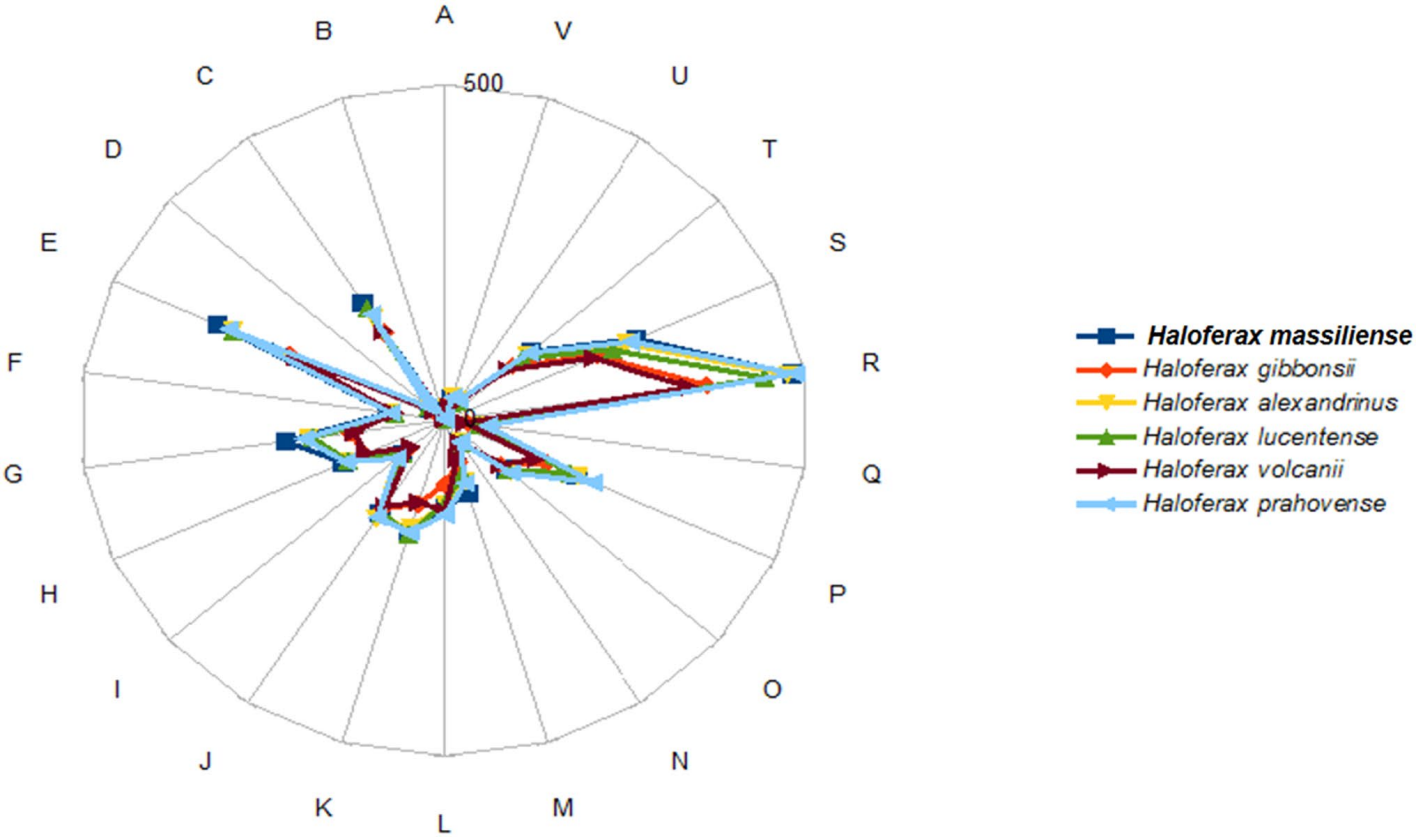
Distribution of functional classes of predicted genes according to cluster of orthologous groups of proteins from *Haloferax massiliense* strain Arc-Hr^T^

The distribution of genes into COG categories was identical (Fig. 6) in all compared genomes.

The Average Genomic Identity of Orthologous gene Sequences (AGIOS) shows that strain Arc-Hr^T^ shared 2.690, 2.353, 2.958, 2.975 and 2.459 orthologous genes with *H. lucentense*, *H. volcanii*, *H. prahovense*, *H. alexandrinus and H. gibbonsii, respectively* (Table 6). Among compared species, except for strain Arc-Hr^T^, AGIOS values ranged from 92.08 to 98.83%. AGIOS values between strain Arc-Hr^T^ and compared species were in the same range (from 92.24% with *H. alexandrinus* to 93.29% with *H. volcanii*). The DDH values ranged from 50.70 to 82.20%, among compared species, except for strain Arc-Hr^T^. Among compared species and strain Arc-Hr^T^, the DDH values ranged from 50.70% with *H. prahovense*, to 54.30% with *H. lucentense,* these values were lower than the 70% cutoff (Meier-Kolthoff et al. 2013) (Table 7).

**Table 6:** Number of orthologous proteins shared between genomes (upper right), average percentage similarity of nucleotides corresponding to orthologous protein shared between genomes (lower left) and number of proteins per genome (bold)

	*H. lucentense*	*H. volcanii*	*H. prahovense*	*H. alexandrinus*	*H. massiliense*	*H. gibbonsii*
*Haloferax lucentense*	4086	2348	2754	2761	2690	2355
*Haloferax volcanii*	97.07	2995	2409	2400	2353	2412
*Haloferax prahovense*	92.1	93.21	4180	3238	2958	2696
*Haloferax alexandrinus*	92.08	92.84	98.83	4109	2973	2685
*Haloferax massiliense*	93.07	93.29	92.33	92.24	4259	2459
*Haloferax gibbonsii*	92.44	93.67	96.68	96.3	92,82	3053

**Table 7:** Pairwise comparison of *Haloferax massiliense* with other species using GGDC, formula 2 (DDH estimates based on identities/HSP length)

	*H. massiliense*	*H. alexandrinus*	*H. gibbonsii*	*H. lucentense*	*H. volcanii*	*H. prahovense*
*H. massiliense*	100 ± 00%	50.90 ± 2.64%	52.90 ± 2.67%	54.30 ± 2.70%	53.70 ± 2.69%	50.70 ± 5.2%
*H. alexandrinus*		100 ± 00%	73.50 ± 2.90%	50. 80 ± 2.63%	53.50 ± 2.68%	96 ± 2.4%
*H. gibbonsii*			100 ± 00%	51.90 ± 2.65%	53.80 ± 2.69%	72 ± 5.8%
*H. lucentense*				100 ± 00%	82.20 ± 2.69%	50.70 ± 5.3%
*H. volcanii*					100 ± 00%	52.80 ± 5.3%
*H. prahovense*						100 ± 00%

The confidence intervals indicate the inherent uncertainty in estimating DDH values from intergenomic distances based on models derived from empirical test data sets (which are always limited in size). These results are in accordance with the 16S rRNA (Fig. 4) and phylogenomic analyses as well as the GGDC results

## Discussion

Here, we describe the genome sequence and most of the biochemical characteristics of the first isolate of *Haloferax massiliense* sp. nov., an extremely halophilic archaea isolated from the human gut. Halophilic organisms are generally known to colonize hypersaline environments where the salt concentration is close to saturation, such as salt lakes and salt marshes (Oren 1994). Here, using a culture medium containing high salt concentration, we successfully isolated strain Arc-Hr^T^ belonging to the *Haloferax* genus within the *Haloferacaceae* family. This strain presents the first halophilic archaea isolated from the human gut. Recently, DNA sequences belonging to some halophilic archaea frequently present or abundant in extreme environments were detected by PCR in the human gastro-intestinal tract as well as some members of the *Halobacteriaceae* family (Oxley et al. 2010). Bacterial halophilism has become a subject of considerable interest for microbiologists and molecular biologists during the past 20 years, because of their development on salty foods (Fukushima et al. 2007). Indeed, these organizations have also been detected in refined salt (Diop et al. 2016) as well as food products where salt is used in large quantities in the process of their conservation such as salted fish, pork ham, sausages and fish sauces (Tanasupawat et al. 2009; Kim et al. 2010). Additionally, the limitation of these organisms to extreme environments has been recently contested after their detection in habitats with relatively low salinity, suggesting an ability of adaptation to survive in more moderate environments (Purdy et al. 2004).

This work does not intend to demonstrate a medical or biotechnological interest regarding strain Arch-Hr^T^; its only aim is to expand knowledge about the human microbiota and isolating all the prokaryotes that colonize the human digestive tract (Lagier et al. 2016).

## Conclusion

Based on the characteristics reported here and the phylogenetic affiliation of strain Arc-Hr^T^, we proposed the creation of *Haloferax massiliense* sp. nov., as a new species belonging to the Haloferax genus with strain Arc-Hr^T^ as its type strain. *Haloferax massiliense* sp. nov., (= CSURP0974 = CECT 9307), described here, was isolated from the human gut as part of a culturomic study aiming at expanding the repertoire of microorganisms colonizing the human gut.

### Description of *Haloferax massiliense* sp. nov

*Haloferax massiliense* (mas.si.li.en’se, N.L. neut. adj., *massiliense* of Massilia, the Roman name of Marseille, France, where the type strain was isolated).

*Haloferax massiliense* strain Arc-Hr^T^ is a strictly aerobic gram negative, non-motile and non-spore-forming. Cells were very pleomorphic (irregular cocci, short and long rods, triangles and ovals) and had a diameter between 1 and 4 µm. An optimal growth was observed at 37 °C, pH 7 and 15% of NaCl. Colonies are red, smooth, shiny and measure 0.5–1 mm. Strain Arc-Hr^T^ has exhibited positive catalase and oxidase activities.

Using API strips, positive reactions were observed for alkaline phosphatase, acid phosphatase, esterase (C4), esterase lipase (C8), leucine arylamidase, naphthol-AS-BI-phosphohydrolase, β-glucuronidase, glycerol, d-fructose, l-rhamnose, potassium 2-ketogluconate and potassium 5-ketogluconate. Strain Arc-Hr^T^ was susceptible to rifampicin and trimethoprim/sulfamethoxazole. The genome of *Haloferax massiliense* is 4,349,774 bp long and exhibits a G + C% content of 65.36%. The 16S rRNA and genome sequences are deposited in EMBL-EBI under accession numbers HG964472 and CSTE00000000, respectively. The type strain Arc-Hr^T^ (= CSUR P0974 = CECT 9307) was isolated from a stool specimen of 22-year-old Amazonian obese female patient as part of a culturomics study.

## Abbreviations

FAME: Fatty acid methyl ester
GC/MS: Gas chromatography/mass spectrometry
CSUR: Collection de Souches de l’Unité des Rickettsies
CECT: Colección Española de Cultivos Tipo
MALDI-TOF MS: Matrix-assisted laser-desorption/ionization time-of-flight mass spectrometry
URMITE: Unité de Recherche sur les Maladies Infectieuses et Tropicales Emergentes
IU: International unit
